# Bacteria enhance the production of extracellular polymeric substances by the green dinoflagellate *Lepidodinium chlorophorum*

**DOI:** 10.1038/s41598-021-84253-2

**Published:** 2021-02-26

**Authors:** Pauline Roux, Raffaele Siano, Karine Collin, Gwenael Bilien, Corinne Sinquin, Laetitia Marchand, Agata Zykwinska, Christine Delbarre-Ladrat, Mathilde Schapira

**Affiliations:** 1 Ifremer, LITTORAL, 44300 Nantes, France; 2 Ifremer, DYNECO, 29280 Plouzané, France; 3 Ifremer, LITTORAL, 29900 Concarneau, France; 4 Ifremer, BRM, 44300 Nantes, France

**Keywords:** Environmental sciences, Ocean sciences, Biogeochemistry, Carbohydrates, Bacteria, Water microbiology, Ecology, Community ecology, Ecophysiology, Microbial ecology

## Abstract

High biomasses of the marine dinoflagellate *Lepidodinium chlorophorum* cause green seawater discolorations along Southern Brittany (NE Atlantic, France). The viscosity associated to these phenomena has been related to problems in oyster cultivation. The harmful effect of *L. chlorophorum* might originate from the secretion of Extracellular Polymeric Substances (EPS). To understand whether the EPS are produced by *L. chlorophorum* or its associated bacteria, or if they are a product of their interaction, batch cultures were performed under non-axenic and pseudo-axenic conditions for three strains. Maximum dinoflagellate cell abundances were observed in pseudo-axenic cultures. The non-sinking fraction of polymers (Soluble Extracellular Polymers, SEP), mainly composed of proteins and the exopolysaccharide sulphated galactan, slightly increased in pseudo-axenic cultures. The amount of Transparent Exopolymer Particles (TEP) per cell increased under non-axenic conditions. Despite the high concentrations of Particulate Organic Carbon (POC) measured, viscosity did not vary. These results suggest that the *L. chlorophorum*-bacteria interaction could have a detrimental consequence on the dinoflagellate, translating in a negative effect on *L. chlorophorum* growth, as well as EPS overproduction by the dinoflagellate, at concentrations that should not affect seawater viscosity.

## Introduction

Green seawater discolorations have been recorded in Southern Brittany (North East Atlantic, France) every year since 1982^1^. These phenomena are the consequences of a massive development of the green dinoflagellate *Lepidodinium chlorophorum*^2,3^, a species characterised by bright green plasts inherited from a secondary endosymbiosis with a chlorophyte^4–6^. *Lepidodinium chlorophorum* is not known to produce toxigenic substances for humans or marine fauna. However, blooms of the species have been associated with mass mortalities of fishes and cultured bivalves^7,8^. The potential harmful effect of *L. chlorophorum* blooms in coastal waters remains to be elucidated, as well as the phenology and physiology of this peculiar dinoflagellate, which have barely been studied so far.

Under laboratory conditions, *L. chlorophorum* excretes a large amount of Transparent Exopolymer Particles (TEP)^9^, which are defined as particles (size > 0.22 μm) stainable with Alcian Blue^10,11^. These particles are the result of TEP-precursor aggregation, mainly composed of exopolysaccharides. The TEP are composed of a large amount of carbon and may play a key role in biogeochemical cycling and in the structure and function of the pelagic food chain^12,13^. Indeed, TEP aggregations tend to accelerate the sedimentation of organic matter from the surface to the seabed^14–16^ and can increase seawater viscosity^11,17^. The TEP accumulations during blooms of *L. chlorophorum* in both the seabed and in the water column may create microenvironments promoting bacterial activity^18–20^, followed by high organic carbon degradations and potential anoxia conditions, likely causing fauna mortalities. As a consequence, the high production of TEP by this bloom-forming dinoflagellate could result in an indirect harmful property of *L. chlorophorum.*

The TEP, together with Soluble Extracellular Polymers (SEP) corresponding to non-sinking fraction of polymers, constitute the Extracellular Polymeric Substances (EPS) produced by some marine microorganisms^21^. The SEP control the floc formation rate and directly influence aggregate formation^11^; it therefore contributes, as TEP, to the modification of seawater viscosity. Beyond *L. chlorophorum*, other species belonging to different phytoplankton classes produce TEP, such as other dinoflagellates^11^, diatoms^11,22–25^, prymnesiophyceae^11,25–27^ and cyanobacteria^28,29^. However, phytoplankton is not the only source of EPS in oceans. Indeed, many studies have demonstrated the exudation of exopolysaccharides by marine bacteria^30–32^. Marine microorganisms produce EPS to promote microbial adhesion^33^ and/or to release metabolic-excess waste products^34^. Therefore, EPS can promote the formation of microalgae aggregates, initiate cell adhesion to a substrate and create biofilm matrix^35^. Furthermore, EPS can protect cells against dewatering and toxic substances and can serve as energy and carbon sinks responding to stress^32^. The EPS produced by microorganisms are mainly composed of exopolysaccharides, proteins (enzymes and structural proteins), nucleic acid (DNA) and lipids^21,32,34^. Humic substances and inorganic components are also found within EPS. Specifically, exopolysaccharides produced by bacteria consist of mannose (Man), rhamnose (Rha), glucose (Glc), galactose (Gal) and galacturonic acid (GalA)^21,32,34^; they are characterised by a high proportion of uronic acid^31^. The protein fraction interacts with polysaccharides and other components to form a stable extracellular matrix. For example, sulphates can generate flocs in the presence of deoxy sugars^36^.

The production and composition of EPS is influenced by various factors including species, strain, substrate type, nutrient availability, environmental conditions (temperature, pH, shear force and salinity), physiology and age of the culture^32^. Laboratory studies have shown a high variability among species in terms of the quantity of EPS produced^9,11,32,37^. For a given species, the amount of TEP produced strongly depends on its environmental growing conditions. Indeed, previous studies have shown an increase in TEP production with an increase in temperature^9,38^, a limitation by nutrients^11,30,39,40^ or an increase in CO_2_ partial pressure^41^. The physiological state of the cells can also strongly condition the quantity of TEP produced, as a close relationship has been established between TEP formation and cell death processes^42^. Moreover, a study suggested the importance of bacteria as mediators of bacteria-associated TEP formation coupled to the supply of usable dissolved organic matter, including TEP precursors^43^. *Lepidodinium chlorophorum* may be listed among the species capable of producing high amounts of TEP^9^. However, the production of TEP by *L. chlorophorum* cultures has been demonstrated only under non-axenic conditions^9^. To the best of our knowledge, no study has investigated the relative contribution, if any, to the EPS production by this dinoflagellate and its associated bacteria. Given that both dinoflagellate and bacteria may produce EPS, it is questionable which microorganism is responsible for EPS production in both natural and cultivated *L. chlorophorum*—bacterial consortia or whether the interaction between both microorganisms can cause an increase in EPS production.

With the aim of identifying the source of EPS production among *L. chlorophorum* and its associated bacteria, we used batch cultures of three strains cultivated under pseudo-axenic (PA) and non-axenic (NA) conditions. We estimated the concentration of TEP at different growth phases under both culture conditions. To further investigate the EPS, for the first time, the SEP was characterised by the contents in proteins, polysaccharides and inorganic compounds, such as sulphur. Finally, we investigated the potential impact of EPS produced by *L. chlorophorum* on viscosity in culture to better understand the consequences of *L. chlorophorum* blooms on ecosystem functioning and potential bloom-associated oyster mortalities.

## Results

### Antibiotic protocol

Our axenization protocol did not result in a complete removal of bacteria from the cultures. This process allowed to drastically reduce the number of bacteria per cell of dinoflagellate, which decreased from 1030 ± 199 in the non-axenic culture (NA) to 104 ± 2 bacteria cell^−1^ after axenization for the strain RCC1489. For *L. chlorophorum* KL1C4, 1639 ± 443 bacteria cell^−1^ were enumerated in the NA culture against only 80 ± 1 bacteria cell^−1^ after axenization. Therefore, 90 and 95% of bacteria per cell could be eliminated via axenization in RCC1489 and KL1C4 cultures, respectively. For *L. chlorophorum* MAR1D2, the number of bacteria per cell decreased from 204 ± 10 in NA conditions to 121 ± 17 bacteria cell^−1^ after axenization. Overall, 41% of bacteria were removed per cell. We therefore assume that our experiments were conducted under pseudo-axenic conditions (PA) (see Supplementary Table S1).

### *Lepidodinium chlorophorum* growth

**Figure 1:**
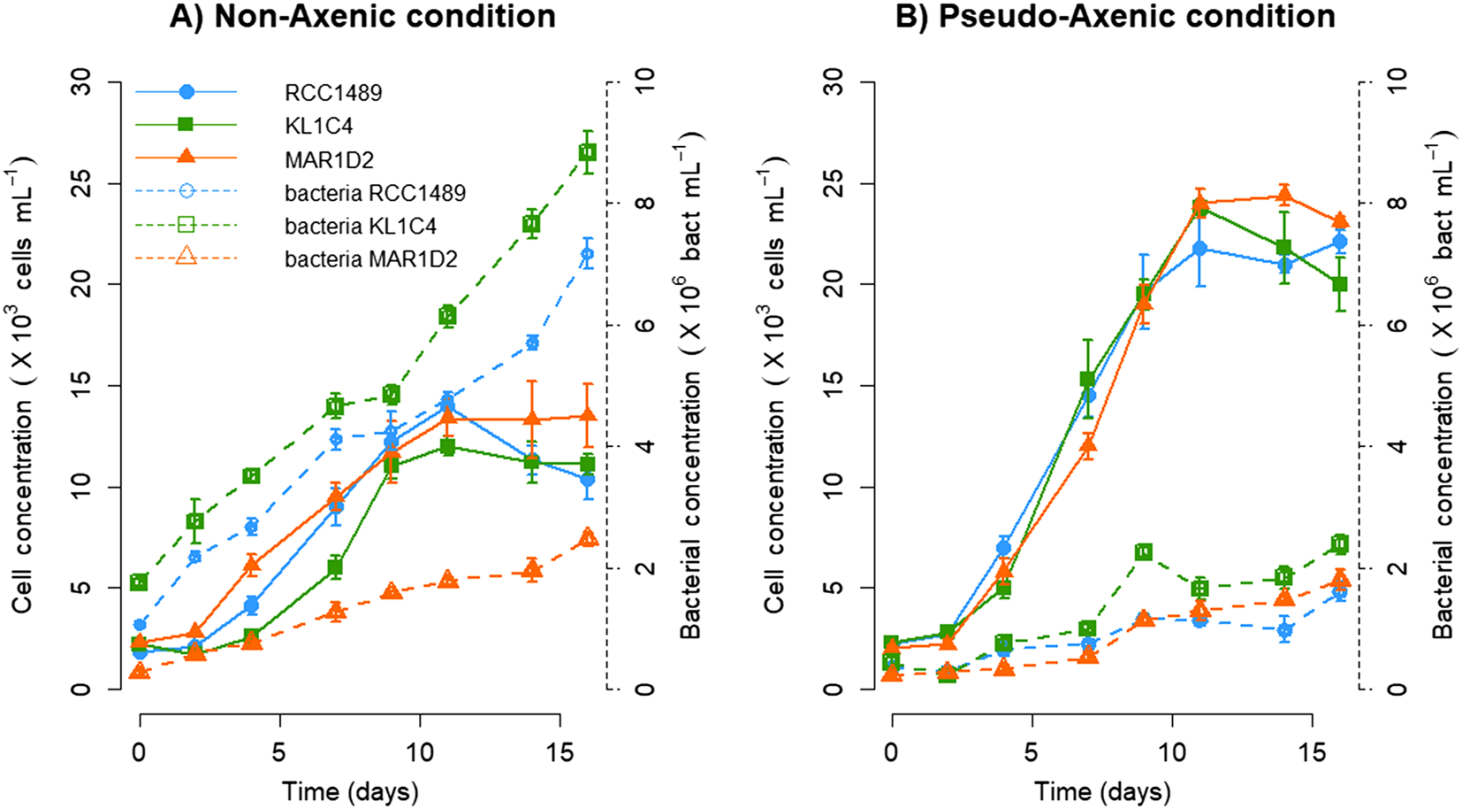
*Lepidodinium chlorophorum* concentrations (cell mL^−1^; solid lines) and bacterial concentrations (bacterial cell mL^−1^; dashed lines) for the three *L. chlorophorum* strains analysed under (**A**) non-axenic (NA) and (**B**) pseudo-axenic (PA) culture conditions. Symbols represent means and error bars represent the standard deviations from triplicate cultures.

*Lepidodinium chlorophorum* growth was analysed for all three strains in both NA and PA culture conditions (Fig. 1). For the NA condition (Fig. 1A), similar maximum *L. chlorophorum* concentrations were observed for RCC1489 (1.4 × 10^4^ ± 646 cells mL^−1^), KL1C4 (1.2 × 10^4^ ± 462 cells mL^−1^) and MAR1D2 (1.4 × 10^4^ ± 1549 cells mL^−1^). For the PA condition (Fig. 1B), the maximum abundance of *L. chlorophorum* was twice as high as that of the NA condition (RCC1489: 2.2 × 10^4^ ± 577; KL1C4: 2.4 × 10^4^ ± 390; MAR1D2: 2.4 × 10^4^ ± 492 cells mL^−1^) (*p* = 0.05 for each strain, one-tailed Wilcoxon signed-rank test). For the NA condition, the maximum bacterial concentration varied between strains, with the MAR1D2 concentration (3 × 10^6^ ± 1 × 10^5^ bacteria mL^−1^) being lower than those of RCC1489 (7 × 10^6^ ± 2 × 10^5^ bacteria mL^−1^) and KL1C4 (9 × 10^6^ ± 3 × 10^5^ bacteria mL^−1^) (Fig. 1A). In PA cultures, bacterial growth slowed down, and maximum bacterial concentrations ($\approx$ 2 × 10^6^ bacteria mL^−1^) remained lower than the minimal values observed in NA for the three strains (Fig. 1). Despite the difference in dinoflagellate cell max and bacterial cell numbers, *L. chlorophorum* growth rates (µ), calculated during the exponential phase under NA and PA conditions, were similar for all strains and conditions, ranging from 0.24 to 0.31 day^−1^. Over time, the maximum quantum efficiency of the photosystem II (F_V_/F_M_) ranged from 0.55 ± 0.00 to 0.72 ± 0.02 (see Supplementary Table S2). These are near-optimal values obtained for chlorophyll *b* containing organisms^44,45^. No significant difference was observed regarding (F_V_/F_M_) values between NA and PA conditions (*p* = 0.8 for RCC1489; *p* = 0.3 for KL1C4; *p* = 0.2 for MAR1D2, two-tailed Wilcoxon signed-rank test). These results indicate a high photosystem II efficiency and, consequently, a good photo-physiological status of the cells throughout the experiment in all culture conditions and for the three studied strains.

### EPS characterisation

**Figure 2:**
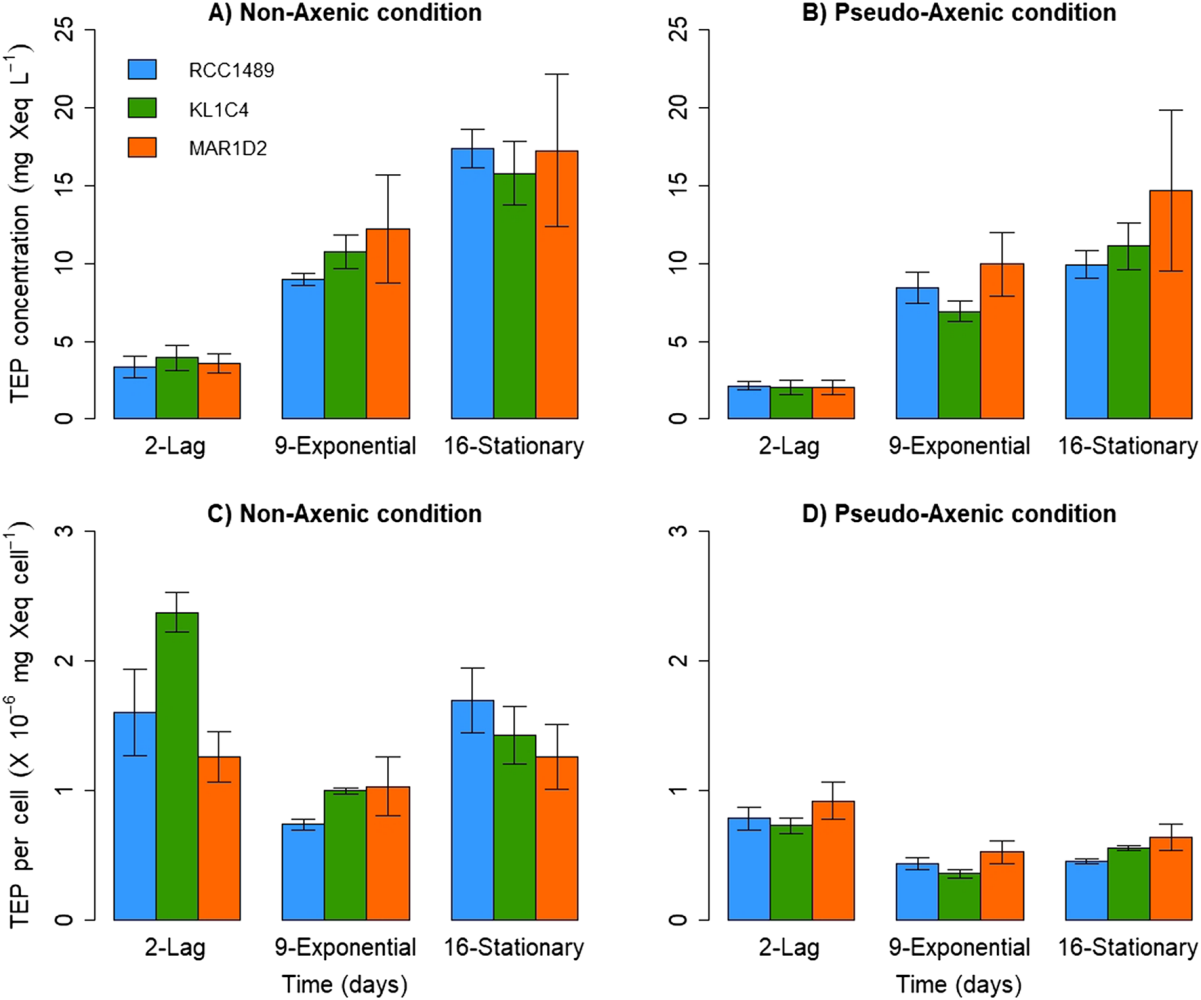
Mean TEP concentrations (mg Xeq L^−1^) measured under (**A**) non-axenic (NA), (**B**) pseudo-axenic (PA) conditions and mean cell normalised TEP production (mg Xeq cell^−1^) estimated under (**C**) NA, (**D**) PA conditions for the three *L. chlorophorum* strains during the different growth phases. Error bars represent standard deviation (n = 3).

For all strains, the TEP concentration increased significantly between the lag and stationary phases (*p* = 0.02 for each strain, two-tailed Kruskal Wallis test) under both NA and PA conditions (Fig. 2). Similar maximum TEP concentrations were observed under the NA condition for RCC1489 (17.4 ± 1.2 mg Xeq L^−1^), KL1C4 (15.8 ± 2.1 mg Xeq L^−1^) and MAR1D2 (17.2 ± 4.9 mg Xeq L^−1^) (Fig. 2A) and under the PA condition (RCC1489: 9.9 ± 0.9; KL1C4: 11.1 ± 1.5; MAR1D2: 14.7 ± 5.1 mg Xeq L^−1^) (Fig. 2B). For all strains, no statistically significant difference was observed regarding TEP concentrations between NA and PA conditions (*p* = 0.1 for RCC1489; *p* = 0.1 for KL1C4; *p* = 0.7 for MAR1D2, two-tailed Wilcoxon signed-rank test) (Fig. 2A,B). This allows to hypothesize that dinoflagellate cells are the main producer of TEP and that bacterial TEP production is negligible. In addition, similar maximal particulate organic carbon (POC) concentrations were observed under the NA condition for RCC1489 (20.4 ± 0.2 mg L^−1^), KL1C4 (18.6 ± 0.5 mg L^−1^) and MAR1D2 (17.5 ± 0.8 mg L^−1^) and under the PA condition (RCC1489: 16.8 ± 0.8; KL1C4: 16.9 ± 0.1; MAR1D2: 16.9 ± 1.1 mg L^−1^) (see Supplementary Table S2). Assuming that majority of TEP production is ascribable to dinoflagellate, we can estimate maximum TEP production per dinoflagellate cell. This value did not vary among strains. Values ranged between 1.3 × 10^–6^ ± 2.5 × 10^–7^ for MAR1D2 and 2.4 × 10^–6^ ± 1.5 × 10^–7^ mg Xeq cell^−1^ for KL1C4 under the NA condition (Fig. 2C) and between 7.3 × 10^–7^ ± 6.2 × 10^–8^ for KL1C4 and 9.2 × 10^–7^ ± 1.4 × 10^–7^ mg Xeq cell^−1^ for MAR1D2 in PA cultures (Fig. 2D). While no significant differences were observed among strains, maximum TEP per cell was significantly higher under NA than under PA for all three strains (*p* = 0.05 for each strain, one-tailed Wilcoxon signed-rank test) (Fig. 2C,D). For the strain RCC1489, TEP production per microalgal cell remained constant over time under both NA and PA conditions. In fact, no significant differences were observed among growth phases (*p* = 0.06, two-tailed Kruskal Wallis test) (Fig. 2C,D). In contrast, for strain KL1C4, TEP per dinoflagellate cell decreased significantly between lag and exponential phases (*p* = 0.02, two-tailed Kruskal Wallis test) under both NA and PA conditions (Fig. 2C,D). A different pattern was observed for strain MAR1D2. While TEP per microalgal cell remained constant over time under NA conditions (Fig. 2C), a significant decrease was observed between lag and exponential phases under PA conditions (*p* = 0.03, two-tailed Kruskal Wallis test) (Fig. 2D).

**Figure 3:**
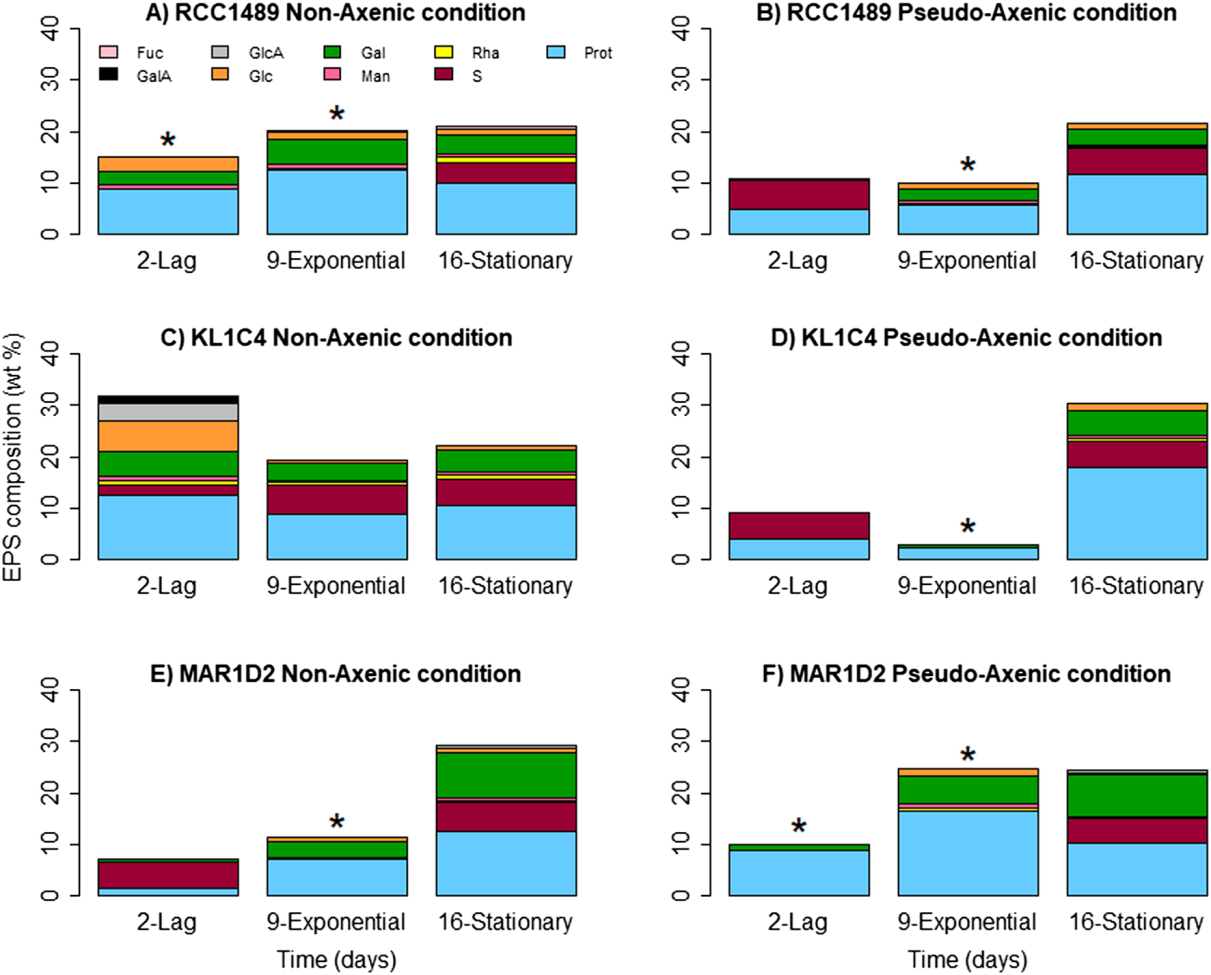
Monosaccharide composition, proteins and sulphate (wt %) of SEP from supernatants, at three growth times, for all three *L. chlorophorum* strains under non-axenic (NA) conditions: (**A**) RCC1489, (**C**) KL1C4, (**E**) MAR1D2 and pseudo-axenic (PA) conditions: (**B**) RCC1489, (**D**) KL1C4, (**F**) MAR1D2 (n = 1). *Prot* proteins, *S* sulphur, *Rha* rhamnose, *Man* mannose, *Gal* galactose, *Glc* glucose, *GlcA* glucuronic acid, *GalA* galacturonic acid, *Fuc* fucose. *Samples were not analysed for their sulphur content due to insufficient sample amount for elementary analysis.

**Figure 4:**
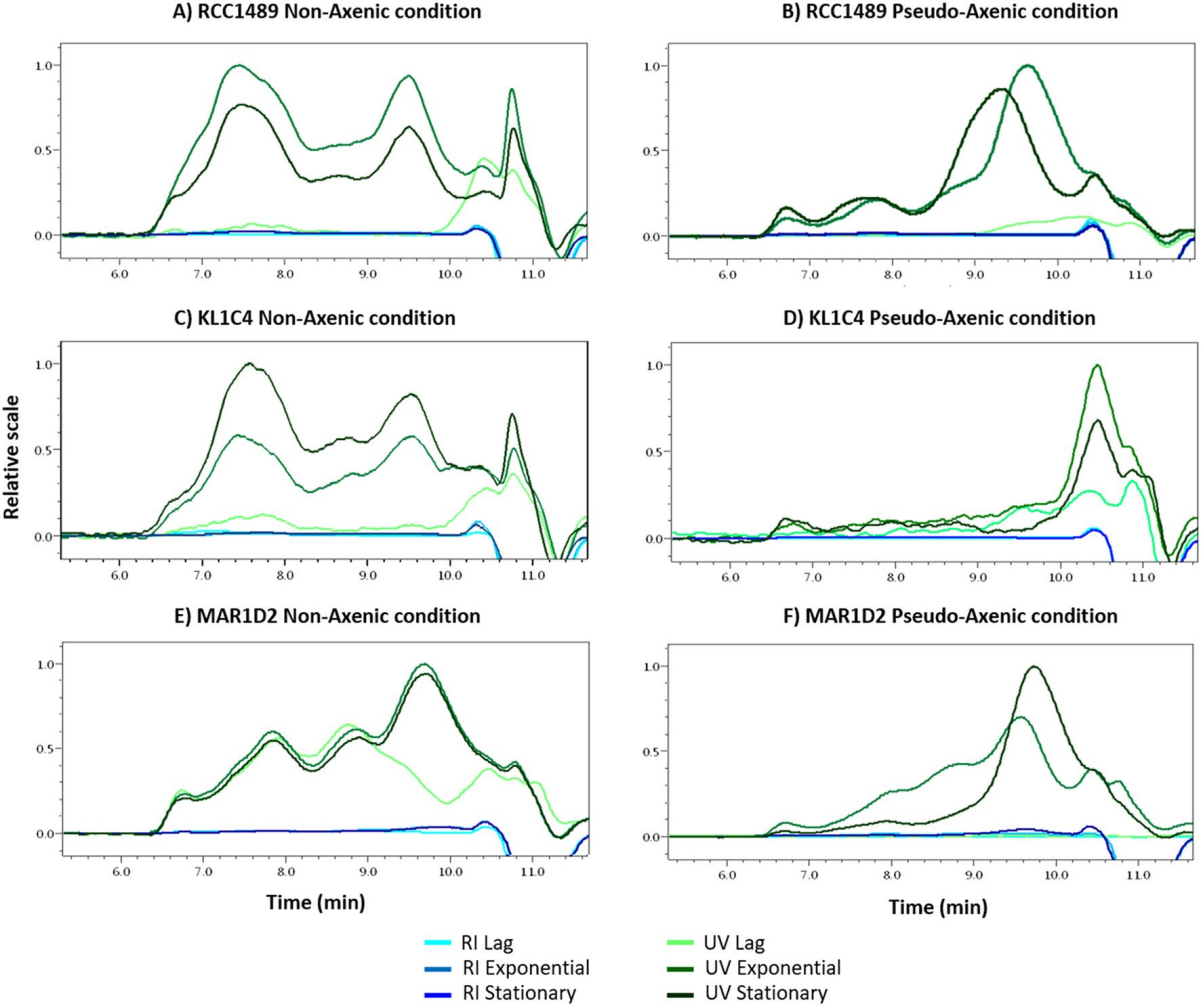
HPSEC profiles (with UV and RI detectors) of culture supernatants obtained for three *L. chlorophorum* strains, at three growth times, under non-axenic (NA) conditions: (**A**) RCC1489, (**C**) KL1C4, (**E**) MAR1D2 and pseudo-axenic (PA) conditions: (**B**) RCC1489, (**D**) KL1C4, (**F**) MAR1D2 [Astra 6.1 Software (WYATT TECHNOLOGY)].

During the stationary phase, SEP yields were higher under PA than under NA conditions for the three strains. For all strains under both NA and PA conditions, SEP yields increased over time except for the strain KL1C4 under NA conditions (Table 1). The SEP yields in *L. chlorophorum* culture supernatants were characterised for protein, sugar and sulphur contents (Fig. 3). The analyses revealed that SEP were mainly composed of proteins (< 20%), followed by neutral sugars (< 10%) and sulphur (3–6%). The composition of SEP was similar for the three strains. Their composition varied only slightly between different growth phases, under both NA (Fig. 3A,C,E) and PA conditions (Fig. 3B,D,F). Amongst neutral sugars, Gal was present in high amounts in SEP of all strains. The highest Gal content was detected in SEP from MAR1D2 in the stationary phase, in both NA (8.9 wt% Gal) and PA (8.2 wt% Gal) conditions. Traces of other neutral sugars such as Glc, Man, Rha and fucose (Fuc) were also detected in several samples. Anionic sugars, glucuronic acid (GlcA) and GalA, were only observed for SEP in the KL1C4 strain under NA conditions (Fig. 3C), and their presence could be explained by contamination of the supernatant by bacterial cell membranes during sample preparation. A similar composition was obtained for SEP closely associated with *L. chlorophorum* cells solubilised from pellets (see Supplementary Table S3). The amount of sulphur detected in the samples was similar for each strain and independent from the growth phase. Sulphur can be associated with sugars and proteins. SEP from RCC1489 and KL1C4 strains were only composed of proteins and sulphur at the lag phase (Fig. 3B,D). To deepen the SEP characterisation under NA and PA conditions, HPSEC-MALS analyses were performed (Fig. 4). Several peaks were observed on HPSEC elution profiles of proteins (followed by UV detector), suggesting the presence of several protein populations (see Supplementary Table S4). Although HPSEC profiles were similar among the three *L. chlorophorum* strains at different growth phases, they clearly varied between NA and PA conditions. Only in NA cultures, protein aggregates of high molecular weight were produced (≥ 1,000,000 g mol^−1^, Supplementary Table S4). Less heterogeneous protein populations were observed in PA cultures. These proteins were mainly of medium and low molecular weights. In contrast to proteins, HPSEC profiles of polysaccharides (RI detector, Supplementary Figure S1) were similar in both culture conditions for the three *L. chlorophorum* strains. Three main peaks were distinguished (see Supplementary Table S4), highlighting the presence of three polysaccharide fractions of high molecular (> 1,000,000 g mol^−1^), medium and low molecular weights. Similar polysaccharides were produced in either NA or PA conditions, with slightly higher amounts under NA. Some protein and polysaccharide peaks eluted at the same time from HPSEC-MALS might suggest the existence of glycoproteins or the presence of proteins and polysaccharides linked together by ionic interactions. To assess whether the polymers are polysaccharides, proteins or glycoprotein conjugates, we analysed SEP components from stationary phases on SDS-PAGE and agarose gel electrophoresis (see Supplementary Figure S2). No difference between NA and PA conditions and between strains was observed. Despite Sypro Ruby sensitive staining for proteins, none of them was observed in the samples. Proteins quantified in the samples by colorimetric assay and HPSEC-MALS could not be revealed by different staining techniques, which leads us to infer that protein aggregates could not enter into the gel and migrate properly prior to be detected by staining. However, electrophoresis allowed to assess the presence of a polysaccharidic fraction that was clearly stained by Stains-All cationic dye. The migration of this polysaccharide in electrophoresis, suggests the presence of sulphated galactose polymer, such as sulphated galactan. Indeed, similar migration patterns were observed for galactan sulphate and dextran sulphate used as references.

**Table 1:** SEP yields (Y; g L^−1^) from culture supernatants for the three *L. chlorophorum* strains during the different growth phases under non-axenic (NA) and pseudo-axenic (PA) conditions.

Condition	*L. chlorophorum* strains					
	RCC1489		KL1C4		MAR1D2	
	NA	PA	NA	PA	NA	PA
**Mean Y (g L**^**−1**^**)**						
Lag	7.8 × 10^–3^	nd	1.8 × 10^–2^	nd	9.9 × 10^–3^	2.2 × 10^–3^
Exponential	8.1 × 10^–3^	5.2 × 10^–3^	1.2 × 10^–2^	1.2 × 10^–2^	8.0 × 10^–3^	6.4 × 10^–3^
Stationary	1.8 × 10^–2^	5.5 × 10^–2^	1.3 × 10^–2^	3.6 × 10^–2^	4.4 × 10^–2^	4.7 × 10^–2^

*nd* not determined because values were overestimated due to the presence of salts.

### Viscosity changes in seawater

Under both conditions, the maximum viscosity excess (ɳ) for all *L. chlorophorum* strains was 2.7% ± 0.4–0.5 (Table 2). No difference was observed between the excess of viscosity under NA and PA conditions during cell growth.

**Table 2:** Mean relative excess viscosity ɳ (%) during each growth phase under non-axenic (NA) and pseudo-axenic (PA) conditions (n = 9) for the three studied *L. chlorophorum* strains (RCC1489, KL1C4 and MAR1D2).

Condition	*L. chlorophorum* strains					
	RCC1489		KL1C4		MAR1D2	
	NA	PA	NA	PA	NA	PA
**Mean ɳ (%)**						
Lag ± S.D	2.1 ± 0.4	0.8 ± 0.4	2.7 ± 0.4	1.6 ± 0.2	0.6 ± 0.5	1.1 ± 0.4
Exponential ± S.D	2.7 ± 0.4	1.6 ± 0.3	1.6 ± 1.1	2.6 ± 0.4	2.7 ± 0.5	0.1 ± 0.2
Stationary ± S.D	2.0 ± 0.4	2.4 ± 1.6	2.0 ± 0.4	2.4 ± 0.8	0.8 ± 0.3	0.8 ± 0.3

### Comparison between non-axenic (NA) and pseudo-axenic (PA) conditions

**Figure 5:**
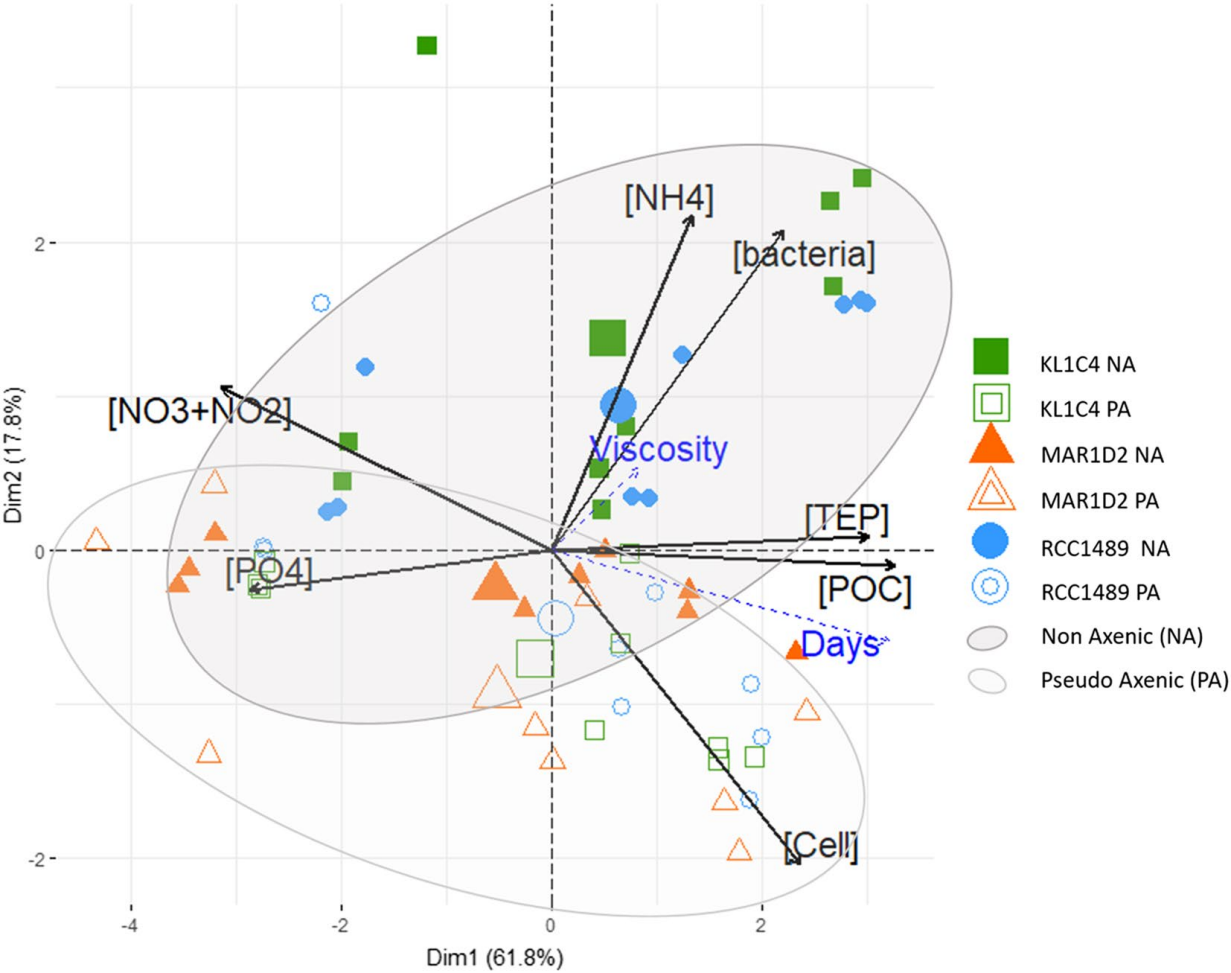
PCA, applied on the dataset (see Supplementary Table S2), summarising the similarities and differences between non-axenic (NA) and pseudo-axenic (PA) samples. Dim1 and Dim2 together describe 79.6% of the total variance. Black arrows are quantitative variables used to calculate PCA: bacterial cell concentration ([bacteria] in bacteria cells mL^−1^: Dim1 = 0.65; Dim2 = 0.61); dinoflagellate cell concentration ([Cell] in cells mL^−1^: Dim1 = 0.70; Dim2 = − 0.60); TEP concentration ([TEP] in mg Xeq L^−1^: Dim1 = 0.89; Dim2 = 0.03); particulate organic carbon concentration ([POC] in mg L^−1^: Dim1 = 0.96; Dim2 = − 0.03); nitrogen ([NO_3_+NO_2_] in µM: Dim1 = − 0.92; Dim2 = 0.31), phosphate ([PO4] in µM: Dim1 = − 0.85; Dim2 = − 0.07) and ammonium concentrations ([NH4] in µM: Dim1 = 0.39; Dim2 = 0.64). Dashed blue arrows are illustrative variables: time ([Days] in numbers) and relative excess viscosity ([Viscosity] in percentage). Strains were represented as follows: RCC1489 (blue circles), KL1C4 (green squares) and MAR1D2 (orange triangle) under NA (filled symbols) and PA conditions (open symbols). Larger symbols (barycentre of each group) and confidence ellipses (95% confidence interval) allowed to distinguish NA (black ellipse) and PA (grey ellipse) conditions.

Principal Components Analysis (PCA), computed on all data collected (see Supplementary Table S2), summarises the differences between NA and PA culture conditions (Fig. 5). The PCA described 79.6% of the total variance along two principal dimensions (Dim1 and Dim2). The Dim1 explained *L. chlorophorum* cell and bacteria concentrations, [TEP] and [POC] as well as [${\text{NO}}_{3}^{-}$ +${\text{NO}}_{2}^{-}$] and [${\mathrm{PO}}_{4}^{3-}$]. [TEP] was positively correlated with [POC], while [${\text{NO}}_{3}^{-}$ +${\text{NO}}_{2}^{-}$] and [${\mathrm{PO}}_{4}^{3-}$] were negatively correlated with *L. chlorophorum* cell, [TEP] and [POC]. In our PCA analysis, $[{\text{NH}}_{4}^{+}]$ was the variable most correlated with Dim2. For strains RCC1489 and KL1C4, PCA provided a clear distinction between samples under NA and PA conditions (Fig. 5). Indeed, NA conditions were positively associated with higher concentrations of bacteria and ammonium, in particular during the stationary phase of *L. chlorophorum* growth. In contrast, PA conditions were positively associated with lower bacterial concentrations and higher *L. chlorophorum* cell concentrations (Fig. 5). [TEP] and [POC] were similar under NA and PA conditions. Relative viscosity excess, used as an illustrative variable, was not correlated with any culture condition. For strain MAR1D2, PCA did not provide a clear distinction between samples under NA and PA conditions because initial bacterial concentrations were lower than those of other strains under NA condition.

## Discussion

Interactions between phytoplankton and heterotrophic bacteria can be mutualistic, competitive or parasitic^46–49^ and can be regulated by algicidal activities^50^. Our results show that similar rates (ranging from 0.24 to 0.31 day^−1^) and any limitation by nutrients characterised the growth of *L. chlorophorum* under both NA and PA conditions. However, *L. chlorophorum* maximum abundances were twice as high in PA than in NA conditions. The antibiotic treatment under PA condition at the beginning of our experiment did not affect the photo-physiological capacities of *L. chlorophorum*, since (F_v_/F_m_) values remained sufficiently high (> 0.55) throughout the experiment without any variation between PA and NA conditions. Indeed, penicillin and streptomycin, used in our treatment, had already shown that an effective bacteriostatic effect did not affect phytoplankton growth^51–54^. We suggest that in our culture conditions, bacteria might have negatively affected *L. chlorophorum* growth, reducing the maximal cell concentrations reachable by our *L. chlorophorum* strains. Indeed, bacteria can strongly affect the growth rate and the maximal biomass reached at the stationary phase of microalgae^55–57^. Guerrini et al*.*^58^ observed an 80% decrease in the cell density of *Cylindrotheca fusiformis* in the presence of bacteria. The algicide effect on microalgae might be caused by the bacterial production of active molecules, as previously reported, such as thermostable benzoic acid produced by the bacterium *Thalassospira* sp*.* ZR-2 against the dinoflagellate *Karenia mikimotoi*^59^, chitinase from *Chitinimonas prasina* LY03 on *Alexandrium tamarense*^60^ and deinoxanthin produced by *Deinococcus xianganensis* Y35, inhibiting the dinoflagellate *A. tamarense*^61^. Furthermore, Lovejoy et al*.*^62^ observed that unarmoured gymnodinoid dinoflagellates (including *Gymnodinium catenatum*) were more sensitive to algicidal bacteria than armoured dinoflagellates (*Alexandrium minutum* and *A. catenella*). For *L. chlorophorum* as well, the absence of theca could facilitate the negative effect of algicidal bacteria. Our study contributes to the understanding of the interactions between dinoflagellate and bacteria, evidencing a complex relationship between the dinoflagellate *L. chlorophorum* and its associated bacterial consortia. However, before concluding on this relationship, varied culture conditions, bacterial isolation and specific observation of the dinoflagellate phycosphere should be investigated.

Total TEP concentrations were similar under both NA and PA conditions for the three *L. chlorophorum* cultivated strains throughout the dinoflagellate growth phases. The SEP yields were higher under PA than under NA conditions. If bacteria had contributed to the EPS production in our culture conditions, we would expect to find higher TEP and SEP concentrations in NA conditions. Hence, our study suggests that *L. chlorophorum* was the main producer of EPS in this dinoflagellate-bacteria consortium. In this study, the maximal TEP concentration produced by *L. chlorophorum* was about 17.4 mg Xeq L^−1^, one of the highest concentrations ever measured in monospecific phytoplankton cultures (Table 3). Moreover, similar maximum TEP concentrations were observed for all strains, suggesting that TEP production was not dependent on intraspecific variability. However, the three strains had been isolated on the French Atlantic coast, while *L. chlorophorum* has been observed in other countries in Europe^63^ as well as in Chile^64^, California^65^ and Australia^66^. Therefore, the variation in TEP production among strains could be due to biogeographical issues. To answer this question, strains from different environments, localities and latitudes should be analysed.

**Table 3:** Maximum TEP concentration (TEPmax; mg Xeq L^−1^) produced by different phytoplankton species grown in cultures (mostly non-axenic).

Species, strain	Class	TEPmax (mg Xeq L^−1^)	References
*Chaetoceros affinis*, CCMP 159	Bacillariophyceae	1.1	Passow^11^
*Chaetoceros* sp., NS isolate		2.8	Passow^11^
*Chaetoceros* sp., UNC 1201		~ 1.1	Burns et al*.*^25^
*Coscinodiscus granii,* AS isolate	Bacillariophyceae	13.2	Fukao et al.^37^
*Skeletonema* sp., AS isolate	Bacillariophyceae	22.4	Fukao et al.^37^
*Eucampia zodiacus,* AS isolate	Bacillariophyceae	16.9	Fukao et al.^37^
*Melosira nummuloides,* NS isolate	Bacillariophyceae	0.3	Passow^11^
*Nitzschia sp.,* SBC isolate	Bacillariophyceae	3.2	Passow^11^
*Rhizosolenia calcar-avis,* CCMP 1518	Bacillariophyceae	0.4	Passow^11^
*Rhizosolenia setigera*, AS isolate		23.5	Fukao et al*.*^37^
*Stephanopyxis turris,* CCMP 815	Bacillariophyceae	9.3	Passow^11^
*Thalassiosira nordenskioeldii*, OW isolate	Bacillariophyceae	1.5	Nosaka et al*.*^24^
*Thalassiosira pseudonana,* CCMP 1335		~ 0.8	Burns et al*.*^25^
*Thalassiosira rotula*, Meunier, NS isolate		0.6	Passow^11^
*Thalassiosira* sp., UNC 1203		~ 0.7	Burns et al*.*^25^
*Thalassiosira weissflogii,* CCMP 1336		0.4	Gärdes et al*.*^23^
*Emiliania huxleyi,* non-calcifying strain, PML 92d	Prymnesiophyceae	0.7	Passow^11^
*Phaeocystis globosa,* CCMP 2754	Prymnesiophyceae	~ 0.5	Burns et al*.*^25^
*Gonyaulax polyedra*, CCMP 406	Dinophyceae	0.3	Passow^11^
*Tetraselmis suecica*, NS isolate	Chlorodendrophyceae	0.9	Passow^11^
*Prochlorococcus marinus,* RCC 0156	Cyanophyceae	1.5	Iuculano et al*.*^29^

*CCMP* Provasoli-Guillard National Center for Culture of Marine Phytoplankton, *PML* Plymouth Marine Laboratory, *UNC* University of North Carolina, *RCC* Roscoff Culture Collection, *NS* North Sea isolate, *SBC* isolated from the Santa Barbara Channel, off California, *AS* Ariake Sound isolate (Japan), *OW* Oyashio Waters (Japan).

In NA conditions, *L. chlorophorum* cell abundances were lower and the TEP amount produced per dinoflagellate cell was higher. This suggests that bacteria, in addition to having an algicidal effect on *L. chlorophorum* growth, induce the overproduction of TEP. In vitro data from this study confirm the observations reported by Passow^11^ that bacteria do not generate significant amounts of TEP in situ, but that high bacterial concentrations may induce high TEP production rates by phytoplankton^67,68^. For example, bacteria may contribute to the generation of TEP by enzymatic hydrolysis of diatom surface mucus^69^. Therefore, bacterial activity may stabilise TEP and increase accumulation rates^69^. In order to establish a carbon footprint in *L. chlorophorum*, TEP produced per cell (mg Xeq cell^−1^) was converted to carbon, following the work of Engel and Passow^70^. These conversions allowed the estimation of the percentage of carbon which was excreted in the form of TEP under NA and PA conditions. Conversion factors of 0.8 and 0.7, respectively, were calculated for NA and PA conditions according to Engel and Passow equations^70^. Under the NA condition, 62–76% of carbon were excreted in the form of TEP for the three strains at day 9 of the cell culture. This result corroborates the percentage found by Claquin et al*.*^9^ who measured, in non-axenic conditions, that for the RCC1489 strain, 70.8% of carbon were excreted as TEP. Under PA conditions, only 43–61% of carbon were excreted as TEP for all three strains. This result suggests that a higher proportion of the carbon fixed by photosynthesis would be allocated to TEP excretion under NA conditions.

For the first time, we characterised SEP produced by *L. chlorophorum*, which were mainly composed of proteins and exopolysaccharides, namely sulphated galactan. The total amount of analysed molecules was limited to a maximum of 30% (w/w) of the dry mass. The analysis of non-purified samples always gives incomplete results. Indeed, gas chromatography analysis of the sugar composition involves a hydrolysis step and a derivatization step to make the compounds volatile. Therefore, the hydrolysis might be incomplete, driving under-evaluated amounts. In addition, the presence of macromolecules in the water-soluble extracts, especially the polyanionic ones, might hinder the total elimination of salt. Nevertheless, galactose represented the main monosaccharide component with proteins and sulphur. The composition of the SEP produced by dinoflagellates is still poorly known. Some studies have focused more specifically on the composition of exopolysaccharides. The presence of Gal residues seems to be a common feature of dinoflagellate exopolysaccharides. Hasui et al*.*^71^ demonstrated that the marine dinoflagellate *Margalefidinium polykrikoides* (cited as *Cochlodinium polykrikoides*) produced sulphated exopolysaccharides mainly composed of Man, Gal, Glc and uronic acid. Yim et al*.*^72^ characterised the exopolysaccharide produced by the marine dinoflagellate *Gyrodinium impudicum* KG03 and found that it was highly sulphated and mainly composed of Gal residues. In 2011, Mandal et al*.*^73^ showed that the toxic dinoflagellate *Amphidinium carterae* produced an exopolysaccharide composed of Gal and Glc residues. Our results suggest that *L. chlorophorum* could also produce a sulphated exopolysaccharide composed mainly of Gal, suggesting that galactose-based exopolysaccharide is a common characteristic among dinoflagellates.

It is questionable whether the high production of EPS, and in particular of TEP, could bring biological and/or ecological benefits to *L. chlorophorum* or if this production is a signal of cellular stress. The TEP production by phytoplankton has been classically described as a consequence of nutrient stress^11,39,40^. However, dinoflagellate cells were not limited by nutrients in our culture condition or in those previously tested on this species^9^. The experimentation in nutrient-depleted cultures is needed to verify if the higher EPS production results from nutrient stress in *L. chlorophorum* culture. In addition, for a given species, the amount of TEP produced strongly depends on other abiotic parameters, especially temperature^9,38^ and partial CO_2_ pressure^41^. Light and temperature were constant during our experiment, and pH did not vary between the two culture conditions, suggesting that the evolution of partial CO_2_ pressure was similar under NA and PA conditions. The mucoid phase could protect *L. chlorophorum* cells against algicidal compounds produced by bacteria. Indeed, EPS can protect cells against toxic substances and can serve as energy and carbon source in stress responses^32^. This hypothesis seems to follow the higher production of TEP per cell that we measured for *L. chlorophorum* in the presence of bacteria. Waiting for new experiments in cellular stress conditions, we can conclude so far that the EPS production by *L. chlorophorum* is a response to a potential algicidal effect of bacteria present in cultures of the dinoflagellate. Nevertheless, in order to validate our hypothesis, culture experiments under axenic condition are needed.

Recent studies have shown that exopolymers produced by phytoplankton can strongly increase the viscosity of seawater^17,74,75^. In situ*,* Seuront et al.^76^ demonstrated that the increase in viscosity ranged from 8.8% before the appearance of *Phaeocystis globosa* to 259% during a bloom of this species. The mucus secreted by *P. globosa* and the subsequent increase in seawater viscosity may be an environmental engineering strategy that *P. globosa* uses to dampen turbulence and to protect colony integrity^77^. In addition, the exudates released by *P. globosa* and the subsequent increase in viscosity might be considered as an antipredator adaptive strategy that ensures the completion of its life cycle in highly turbulent environments^77^. Therefore, biologically increased seawater viscosity might have significant impacts on a range of ecological processes^78^. Indeed, TEP provide physical structure to microhabitats by retaining trace elements and organic-rich matter. They may also act as barriers to diffusion and create patchiness in chemical properties. These microzones would affect the chemotactic behaviour of protozoa and their predation rate^11^. Despite the high TEP concentrations measured in *L. chlorophorum* cultures, no increase in viscosity was observed under any condition tested. The physicochemical properties of EPS are attributed to their diverse and complex chemistry and change with species diversity, age and growth conditions^21^. Physical factors, such as turbulence regime, may have an impact on the formation and persistence/dispersal of TEP in situ, as shear enhances the coagulation of TEP-precursors^11,69,79^ and can modify seawater viscosity. Our culture conditions were carried out in a steady turbulence regime, and thus, our experiments can be hardly extrapolated to in situ conditions. We can neither conclude on the effect of turbulence on TEP production in situ nor on the potential ecological impact and effect of this production on bloom phenology. Measurements of seawater viscosity and bacterial concentration during a bloom of *L. chlorophorum* are needed to verify the experimental hypothesis advanced in this study.

In situ analyses could also contribute to elucidate the effect of excreted TEP on bivalves. Indeed, TEP aggregations tend to accelerate the sedimentation of organic matter from the surface to the seabed^14–16^. The rapid sedimentation of this high quantity of organic carbon could accentuate hypoxia and therefore contribute to the mortalities of natural or cultivated bivalve populations. The large amount of TEP excreted by *L. chlorophorum* could also enhance remineralisation processes in the water column and close to the water–sediment interface. As shown in our study, TEP production was associated with high POC concentrations. In addition, this dinoflagellate is not an edible prey by oysters on the basis of Dynamic Energy Budget (DEB) modelling^80,81^. It remains to be demonstrated that the non-palatability of this prey by oyster could depend on TEP production. In situ coupled to in vitro experiments focused on the interaction between *L. chlorophorum* and oysters could complete the analyses on the ecological and eventually harmful impact of this dinoflagellate. Beyond our specific case, this study could provide an example of how an environmental impact could be addressed integrating cellular biology, physiological and ecological approaches.

## Methods

### Microalgal strains and culture conditions

Three strains of *L. chlorophorum* were used in this study: (1) RCC1489 (RCC: Roscoff Culture Collection; http://roscoff-culture-collection.org/) isolated in the Seine Bay (Normandy, France) in 2005, (2) KL1C4 (IFR CC 18-001, RCC6910) isolated in the Douarnenez Bay (Northern Brittany, France) in 2018 and (3) MAR1D2 (IFR CC 19-001, RCC6911) isolated in the Vilaine Bay (Southern Brittany, France) in 2019. The three strains were maintained in culture in L1 medium^82^ without Si, at 20 °C under a 12:12 h light: dark cycle, with 90 μmol photon m^−2^ s^−1^ illumination.

All strains were genetically identified by Sanger sequencing using the Large Sub Unit (LSU) (28S) region of the ribosomal DNA (rDNA). Extraction of *L. chlorophorum* strains DNA and PCR amplification were carried out using the PCRBIO Rapid Extract PCR Kit (PCR BIOSYSTEMS LDT. London). The DNA was extracted from a 20-µL aliquot at the exponential growth phase following the manufacturer´s recommendations, except for the dilution step, where 190 μL of nuclease-free water (instead of 900 μL) were added in the protease deactivation step. The PCR was performed using primers D1R-D3B^83,84^. The PCR cycling comprised an initial 2 min heating step at 95 °C, followed by 40 cycles of 95 °C for 15 s, 56 °C for 15 s and an extension at 72 °C for 30 s. The PCR-amplified products were analysed on a 1% agarose TAE gel (ethidium bromide; BET 1X) and purified using the ExoSAP-IT PCR Product Cleanup reagent (AFFYMETRIX, Cleveland, OH, USA). The Big Dye Terminator v. 3.1 Cycle Sequencing Kit (APPLIED BIOSYSTEMS, Tokyo, Japan) and ABI PRISM 3130 Genetic Analyzer (APPLIED BIOSYSTEMS) were used for amplicon sequencing. Sequences were verified on the National Center for Biotechnology Information website (https://www.ncbi.nlm.nih.gov/) using Nucleotide Blast. Sequences of strains KL1C4 and MAR1D2 were deposited on GenBank under the accession numbers MT850080 and MT850081.

### Antibacterial protocol

To reduce the abundance of bacteria in *L. chlorophorum* cultures, an antibacterial protocol was applied as follows. For each strain, 40 mL of cultures were sampled at the exponential growth phase. Samples were gently filtered through 3-µm polycarbonate membrane filters (WHATMAN Nuclepore Track-Etched Membrane). The filtrates were discarded and the cells rinsed with 40 mL sterile L1 medium. The solution containing the cells was then centrifuged (1000*g* for 10 min at 20 °C). This washing step was repeated three times. Finally, the pellets were re-suspended in sterile L1 medium for further axenization. To eliminate epiphytic bacteria of the dinoflagellate cell wall, samples were incubated with Tween-80 (0.005%) at 20 °C for 10 min. Thereafter, samples were washed twice in a row with sterile L1 medium. Finally, based on a specific mix of antibiotics (X100, CORNING, 30-002-CI), 100 IU penicillin and 100 µg streptomycin per mL of culture were added. To confirm the effect of this axenization protocol on *L. chlorophorum* cultures, heterotrophic bacteria were enumerated following Marie et al*.*^85^, using an Accuri C6 flow cytometer (BECTON DICKINSON) equipped with blue (488 nm) and red (640 nm) lasers, detectors of forward (FSC) and side (SSC) light scatter, and four fluorescence detectors: 530 ± 15 nm (FL1), 585 ± 20 nm (FL2), > 670 nm (FL3) and 675 ± 12.5 nm (FL4). Briefly, bacteria were stained with SYBR Green I, then counted based on plots of red fluorescence versus green fluorescence and of side scatter versus green fluorescence.

### Experimental set-up for *L. chlorophorum* cultures

The three strains of *L. chlorophorum* were cultivated for 16 days in non-axenic (NA) and pseudo-axenic (PA) batch cultures at 20 °C under a 12:12 h light: dark cycle, with 90 μmol photon m^−2^ s^−1^ illumination. For PA cultures, antibiotics were added at the beginning of the experiment (day 0) to maintain the lowest abundance of bacteria during the experiment*.* Nine 500 mL flasks were inoculated with 2000 cells mL^−1^ for each strain under both NA and PA conditions. In three of these flasks, subsamples were collected every 2 days to evaluate *L. chlorophorum* and bacterial concentrations over time. At three selected phases of cellular growth (lag phase on day 2, exponential growth on day 9 and stationary phase on day 16), culture subsamples were collected from three flasks per growth phases. This strategy was chosen to obtain sufficient biomass allowing analyses in triplicate of maximum photochemical efficiency of the photosystem (II), inorganic nutrients concentrations, POC and TEP concentrations and viscosity. Analyses of SEP were performed on the pooled biomass remaining from triplicate samples of each growth phase.

### Biological, biochemical and chemical analyses

To quantify *L. chlorophorum* cells, 1.5-mL triplicates were fixed with acidic Lugol’s solution (1% final concentration) and stored at 4 °C until analysis. Cell enumeration was performed in a 1-mL Sedgewick Rafter Counting Cell under an inverted microscope (LEICA DMI3000B). Growth rate (µ) was calculated during exponential growth using the least-squares regression method^86^. For bacterial cell determination, 1-mL triplicates were fixed with glutaraldehyde (0.1% final concentration) (SIGMA-ALDRICH) and stored at -80 °C until analysis. Enumeration was carried out using an Accuri C6 flow cytometer.

The photo-physiological status of *L. chlorophorum* cells during the experiment was verified by measuring the maximum quantum efficiency of the photosystem II (Fv/Fm) at 450 nm, using an Aquapen-C 100 fluorimeter (PHOTON SYSTEMS INSTRUMENTS). Three mL triplicates were maintained in the dark for 15 min, following standard protocols^87^, before measurements.

To follow nutrient concentrations of *L. chlorophorum* cultures during the experiments, 7 mL (× 2) were sampled from three flasks, for three different growth phases, and stored directly at − 20 °C for the determination of dissolved inorganic nitrogen (i.e. N = ${\text{NO}}_{3}^{-}$+${\text{NO}}_{2}^{-}$ +${\text{NH}}_{4}^{+}$) and phosphate concentrations (${\mathrm{PO}}_{4}^{3-})$. Triplicates were analysed with an auto-analyser (SEAL ANALYTICAL AA3) following standard protocols^88^. Limits of quantification were 0.5 µM for ${\text{NO}}_{3}^{-}$+${\text{NO}}_{2}^{-}$, 0.05 µM for ${\text{NO}}_{2}^{-}$, 0.05 µM for ${\mathrm{PO}}_{4}^{3-}$ and 0.05 µM for ${\text{NH}}_{4}^{+}$.

### EPS determination: TEP and SEP analyses

For the three different growth phases, the concentration of TEP was determined using a semi-quantitative method based on the colorimetric determination of the amount of dye complexed with extracellular particles (Claquin et al.^9^ adapted from Passow and Alldredge^22^). Culture subsamples of 5–20 mL were gently filtered through 0.4 µm polycarbonate membrane filters (WHATMAN Nuclepore Track-Etched Membrane) and stored at − 20 °C until analysis. Particles retained on the filters were stained with 5 mL of 0.02% Alcian blue (SIGMA) in 0.06% acetic acid (pH 2.5). Alcian blue is a hydrophilic cationic dye that complexes anionic molecules bearing negative substituents such as carboxyl or sulphate groups. After centrifugation at 4000*g* for 30 min, supernatants were removed and filters were further centrifuged several times with 5 mL of MilliQ water until all excess dye was removed from the pellet. After one night of drying at 50 °C, 6 mL of 80% H_2_SO_4_ were added, and 2 h later, the absorption of the supernatant was measured using a spectrometer at 787 nm (SHIMADZU UV-2600). Alcian blue absorption was calibrated using a solution of Xanthan gum (X). The TEP concentrations are expressed in mg Xeq L^−1^.

To characterise SEP, approximately 900 mL of culture biomass were centrifuged (4000*g* for 30 min at 20 °C). Supernatants were concentrated and desalted by using an ultra-filtration system (PELLICON MILLIPORE) with a 5-kD cut-off membrane and freeze-dried. Pellets were fixed for 1 h at 20 °C with a solution of 5% formol/ethanol (w/w), dialysed against water (3.5 kD porous membrane) to eliminate salts and freeze-dried. Pellets were then solubilised in water for 1 h at 60 °C to recover SEP eventually associated with *L. chlorophorum* cells. Supernatants recovered after centrifugation (4000*g* for 15 min) were freeze-dried. Prior to analyses, all samples were solubilised in water at 3 mg/mL. The protein content was estimated according to the bicinchoninic-acid protein assay (BCA), and Bovine Serum Albumin was used as standard^89^. Monosaccharide composition was determined by gas chromatography (GC) analysis of trimethylsilyl derivatives after acid methanolysis^90^. Briefly, supernatants were hydrolysed using MeOH/HCl for 4 h at 100 °C. Myo-inositol was used as internal standard. The methyl glycosides thus obtained were then converted to trimethylsilyl derivatives using *N*,*O*-bis(trimethylsilyl)trifluoroacetamide and trimethylchlorosilane (BSTFA:TMCS) 99:1 (MERCK). Gas chromatography (GC-FID, AGILENT TECHNOLOGIES 6890N) was used for separation and quantification of the per-*O*-trimethylsilyl methyl glycosides formed. The sulphur content was determined by elementary analysis performed at the BioCIS-UMR 8076 (Châtenay-Malabry, France). The molecular weights of proteins and polysaccharides were determined by HPSEC (High Performance Size-Exclusion Chromatography, HPLC Prominence SHIMADZU Co, Kyoto, Japan) coupled on-line with a multiangle light scattering detector MALS (Dawn Heleos-II, WYATT TECHNOLOGY, Santa Barbara, CA, USA), a differential refractive detector (RI) (Optilab WYATT TECHNOLOGY, Santa Barbara, CA, USA) and a UV detector at 280 nm. Samples with concentrations from to 2 to 3 mg mL^−1^ were solubilised and filtered on a 0.45 µm syringe filter; 100 µL were injected on a PL aquagel-OH mixed, 8 μm (AGILENT) guard column (U 7.5 mm × L 50 mm), and a PL aquagel-OH mixed (AGILENT) separation column. Elution was carried out at 1 mL min^−1^ with 0.1 M ammonium acetate. The chromatogram was further processed with Astra 6.1 Software (WYATT TECHNOLOGY). The refractive index increments used were dn/dc = 0.145 mL g for polysaccharides and 0.185 mL g for proteins. Polyacrylamide and agarose gel electrophoresis was applied to refine the polymer composition. The SDS-PAGE with a 10% polyacrylamide separating gel was prepared following the common method of Laemmli^91^. Briefly, 25 µL of each sample were loaded on a gel; 1% agarose gel was prepared in TAE (40 mM Tris base, 1.1 mL/L acid acetic, 2 mM EDTA). Samples were mixed 4:1 (v/v) with a sample loading buffer (0.5 M Tris HCl pH 6.8, glycerol, 0.5 M EDTA, 0.5% w/v bromophenol), and 10 µL of the sample were loaded onto a gel. Gels were run in TAE buffer at 150 V. After migration, gels were stained with Stains-All to detect anionic polysaccharides as previously described^92^. Carbohydrates were also revealed by Schiff staining^93^, while proteins were detected by Sypro Ruby staining (Sypro Ruby protein gel stain, INVITROGEN). The *Escherichia coli* strain O111:B4 lipopolysaccharide, Bovine Serum Albumin, and different polysaccharides (galactan sulphate with 7.7% sulphur content, dextran sulphate sodium salt from *Leuconostoc* spp. (MW 500 000 with 16.0–19.0% sulphur and MW 50 000 with 16.0–19.0% sulphur; SIGMA-ALDRICH, Saint Quentin Fallavier, France)) were used as references.

### POC determination

To estimate the amount of particulate carbon due to the entire TEP produced by *L. chlorophorum* and/or associated bacteria, 10–20 mL triplicates (from three different flasks) were gently filtered onto combusted GF/F filters (WHATMAN Nuclepore; for 4 h at 450 °C) and stored at − 20 °C until analysis. After removal of carbonates with phosphoric acid^94^, filters were treated using a CHN element analyser (Flash 2000, THERMO FISHER SCIENTIFIC, USA) to measure POC concentration. To estimate the carbon enrichment due to antibiotics addition in Pseudo-Axenic (PA) cultures, POC concentration was measured on three replicates of L1 medium with antibiotics and the mean value was subtracted.

### Viscosity measurements

Viscosity measurements were conducted on 2-mL triplicate samples using an Ubbelohde viscometer and following the procedure described by Seuront et al*.*^77^. The measured viscosity *η*_*m*_ (cP) is the sum of a physically controlled viscosity component *η*_*T,S*_ (cP) and a biologically controlled viscosity component *η*_*Bio*_ (cP):

1 $${\eta }_{m}={\eta }_{T,S}+{\eta }_{Bio}$$

For non-axenic and pseudo-axenic conditions, the physically controlled components η_T,S_ were estimated from viscosity measurements conducted respectively on sterile L1 medium or sterile L1 medium with antibiotics.

The biologically induced viscosity excess η_Bio_ (cP) was subsequently defined for each water sample as:

2 $${\eta }_{Bio}={\eta }_{m}-{\eta }_{T, S}$$

The relative viscosity excess η (%) was then calculated as follows:

3 $$\eta = \frac{({\eta }_{m }-{\eta }_{T,S })}{{\eta }_{T,S}}$$

Viscosity measurements were conducted at constant temperature. A thermometer (DOSTMANN ELECTRONIC P655) and conductometer (HACH CDC401) were used to measure temperature and salinity, respectively.

### Statistical analyses

Data are presented as triplicate means with standard deviation (S.D.). The number of samples per group of variables was small (n < 10), which implies that the hypotheses of normal distribution (Shapiro–Wilk test) and homoscedasticity of residuals (Bartlett test) were not verified. Therefore, non-parametric tests of Kruskal–Wallis and post hoc multiple comparison were carried out to check the statistical significance of the differences observed among the data of the three strains and the three different growth phases. Wilcoxon signed-rank test was applied to check differences between non-axenic (NA) and pseudo-axenic (PA) conditions. Statistical analyses were performed using the R software^95^. A Principal Components Analysis (PCA), conducted with the *FactoMine*R package^96^, was applied to assess differences and similarities between samples under NA and PA conditions. Dinoflagellate and bacterial cell abundances, concentrations of TEP and POC as well as nutrient concentrations were used as quantitative variables. Time (in days) and excess of viscosity (in percentage) were used as illustrative variables. The representation of confidence ellipses (95% confidence interval) around the barycentre of each condition allowed to distinguish data group.

## Supplementary Information

Supplementary Information
