# Supplementary material for: Bacteria enhance the production of extracellular polymeric substances by the green dinoflagellate Lepidodinium chlorophorum
Source: Sci Rep. 2021 Feb 26;11:4795. doi: 10.1038/s41598-021-84253-2 (PMC7910647; doi:10.1038/s41598-021-84253-2)
Supplement: Supplementary file 1 — Supplementary Information [file 41598_2021_84253_MOESM1_ESM.pdf]

Supplementary Information

Bacteria enhance the production of extracellular polymeric substances by the green dinoflagellate *Lepidodinium chlorophorum*

Pauline Roux, Raffaele Siano*, Karine Collin, Gwenael Bilien, Corinne Sinquin, Laetitia Marchand, Agata Zykwinska, Christine Delbarre-Ladrat and Mathilde Schapira

*raffaele.siano@ifremer.fr

Supplementary Table S1. Mean bacteria per *L. chlorophorum* cell (mean ± s.d.) enumerated during the three different growth phases (lag: "Lag"; exponential: "Expo."; stationary: "Stat.") for the three different strains (RCC1489, KL1C4 and MAR1D2) in non-axenic (NA) and pseudo-axenic (PA) conditions. Mean and standard deviation values were calculated from the values measured in the three replicates (n = 3). The percentage of axenization was calculated with the following formula: $Axenization\ (\%) = 100 - (\frac{\mathrm{PA}}{\mathrm{NA}} * 100\ )$.

Strain	Time (days)	Growth phase	NA (bacteria cell^{-1})	PA (bacteria cell^{-1})	Axenization (%)
RCC1489	2	Lag	1030±199	104±2	90
	9	Expo.	348±19	59±10	83
	16	Stat.	697±84	71±4	90
KL1C4	2	Lag	1639±443	80±1	95
	9	Expo.	450±43	115±6	74
	16	Stat.	795±40	119±2	85
MAR1D2	2	Lag	204±10	121±17	41
	9	Expo.	136±16	60±5	56
	16	Stat.	184±23	78±7	58

Supplementary Table S2. Mean parameters (mean ± s.d.) measured during the three different growth phases (lag: "Lag"; exponential: "Expo."; stationary: "Stat.") for the three different strains (RCC1489, KL1C4 and MAR1D2) in non-axenic and pseudo-axenic conditions. Mean and standard deviation were calculated from the values measured in the three replicates (n = 3). Cell concentration (*L. chlorophorum*; cells mL^{-1}); Bacterial concentration (Bacteria; bact. mL^{-1}); Maximum quantum yield of the photosystem II (Fv/Fm); Transparent exo-polymers particles concentration (TEP; mg Xeq L^{-1}); Particulate organic carbon concentration (POC; mg L^{-1}); Nitrate (NO_3^-), Nitrite (NO_2^-), Ammonium (NH_4^+) and Phosphorus (PO_4^{3-}) concentrations are expressed in µM; Relative excess of viscosity (Viscosity; %).

Strains	Growth phase	*L. chlorophorum* (cells mL^{-1})	Fv/Fm (450 nm)	Bacteria (bact. mL^{-1})	TEP (mg Xeq L^{-1})	POC (mg L^{-1})	NO_3^- (µM)	NO_2^- (µM)	NH_4^+ (µM)	PO_4^{3-} (µM)	pH	Viscosity (%)
**NON-AXENIC**												
RCC1489	Lag	2 10^3±5 10^2	0.68±0.01	2 10^6±7 10^4	3.4±0.7	6.2±0.2	868.5±1.8	2.5±0.0	0.3±0.0	34.0±0.0	8.1±0.0	2.1±0.4
	Expo.	12 10^3±4 10^2	0.66±0.02	4 10^6±3 10^5	9.0±0.4	14.3±1.3	725.4±4.4	5.2±0.3	1.5±0.7	27.3±0.6	8.7±0.1	2.7±0.4
	Stat.	10 10^3±1 10^3	0.65±0.01	7 10^6±2 10^5	17.4±1.2	20.4±0.2	663.8±5.1	10.2±3.4	1.8±0.2	24.7±0.6	8.7±0.0	2.0±0.4
KL1C4	Lag	2 10^3±3 10^2	0.65±0.01	3 10^6±3 10^5	3.9±0.8	6.4±0.2	882.0±1.0	4.0±0.0	0.4±0.0	34.3±0.6	8.1±0.0	2.7±0.4
	Expo.	11 10^3±6 10^2	0.64±0.01	5 10^6±1 10^5	10.8±1.1	14.3±0.7	794.1±1.1	4.9±0.0	0.8±0.3	30.0±0.0	8.5±0.0	1.6±1.1
	Stat.	11 10^3±5 10^2	0.61±0.01	9 10^6±3 10^5	15.8±2.1	18.6±0.5	692.8±0.6	5.9±0.2	1.9±0.4	26.0±0.0	8.6±0.0	2.0±0.4
MAR1D2	Lag	2 10^3±3 10^1	0.72±0.02	6 10^5±2 10^4	3.5±0.6	6.6±0.4	931.5±4.2	0.9±0.1	0.4±0.2	50.7±2.3	8.2±0.0	0.6±0.5
	Expo.	12 10^3±2 10^2	0.68±0.01	2 10^6±2 10^4	12.2±3.5	13.5±1.4	778.5±4.0	6.1±0.2	1.0±0.4	32.7±0.6	8.6±0.0	2.7±0.5
	Stat.	14 10^3±2 10^3	0.55±0.00	3 10^6±1 10^5	17.2±4.9	17.5±0.8	669.3±5.1	9.4±1.4	0.9±0.2	29.7±0.6	8.7±0.1	0.8±0.3
**PSEUDO-AXENIC**												
RCC1489	Lag	2 10^3±9 10^1	0.71±0.01	3 10^5±6 10^3	2.1±0.3	2.7±0.2	886.9±11.3	1.6±0.0	0.5±0.0	34.7±0.6	8.2±0.1	0.8±0.4
	Expo.	20 10^3±2 10^3	0.67±0.01	1 10^6±9 10^4	8.4±1.0	12.2±0.4	700.7±1.9	4.3±2.7	1.5±0.5	28.0±0.0	8.7±0.1	1.6±0.3
	Stat.	22 10^3±6 10^2	0.62±0.01	2 10^6±1 10^5	9.9±0.9	16.8±0.8	583.3±8.3	7.1±0.1	1.2±0.5	25.7±0.6	8.6±0.1	2.4±1.6
KL1C4	Lag	2 10^3±1 10^2	0.70±0.01	2 10^5±5 10^3	2.0±0.4	3±0.1	880.4±5.1	1.6±0.0	0.4±0.0	35.0±0.0	8.2±0.0	1.6±0.2
	Expo.	20 10^3±8 10^2	0.64±0.01	1 10^6±9 10^4	7.0±0.7	12.2±0.3	702.2±5.1	4.8±0.2	1.6±0.5	30.0±0.0	8.7±0.0	2.6±0.4
	Stat.	20 10^3±1 10^3	0.62±0.01	2 10^6±1 10^5	11.1±1.5	16.9±0.1	598.4±2.5	6.6±0.2	0.6±0.4	28.0±0.0	8.6±0.0	2.4±0.8
MAR1D2	Lag	2 10^3±2 10^2	0.71±0.01	3 10^5±3 10^4	2.0±0.5	1.4±0.2	946.8±9.0	2.6±0.0	0.6±0.3	48.0±8.5	7.9±0.0	1.1±0.4
	Expo.	19 10^3±9 10^2	0.69±0.01	1 10^6±4 10^4	10.0±2.1	10.6±0.6	754.1±6.0	5.9±0.3	0.9±0.8	33.0±0.0	8.7±0.0	0.1±0.2
	Stat.	23 10^3±3 10^2	0.67±0.01	2 10^6±1 10^5	14.7±5.1	16.9±1.1	597.1±16.8	13.0±1.3	0.8±0.6	30.0±1.0	8.7±0.0	0.8±0.3

Supplementary Table S3. Soluble Extracellular Polymers (SEP) from pellets characterized during the three different growth phases (lag: "Lag"; exponential: "Expo."; stationary: "Stat.") for the three different strains (RCC1489, KL1C4 and MAR1D2) in non-axenic and pseudo-axenic conditions (n = 1). Monosaccharide composition, proteins and sulphur are expressed in percentage of weight (wt %). Prot: Proteins; Rha: Rhamnose; Fuc: Fucose; Man: Mannose; Gal: Galactose; Glc: Glucose; GlcA: Glucuronic acid; GalA: Galacturonic acid; S: Sulphur; nd: not determined.

Sample (pellet)	Time (days)	Growth phase	Prot (wt %)	Rha (wt %)	Fuc (wt %)	Man (wt %)	Gal (wt %)	Glc (wt %)	GlcA (wt %)	GalA (wt %)	S (wt %)
**NON-AXENIC**											
RCC1489	2	Lag	nd	nd	nd	nd	nd	nd	nd	nd	nd
	9	Expo.	11.8	0	0	0.2	1.4	0.3	0	0	2.7
	16	Stat.	5.7	0.3	0	0.2	1.9	0.6	0	0	2.9
KL1C4	2	Lag	11.8	0	0	0	0.3	0.3	0	0	nd
	9	Expo.	8.2	0.4	0	0.3	2.9	0.7	0	0	3.6
	16	Stat.	5.1	0.3	0	0.2	2	0.4	0	0	3.3
MAR1D2	2	Lag	4.7	0	0	0	1.1	0	0	0	nd
	9	Expo.	14.2	0	0	0	3.5	0.5	0	0	3.1
	16	Stat.	7.9	0	0	0	2.3	0.2	0	0	4.7
**PSEUDO-AXENIC**											
RCC1489	2	Lag	4.1	0	0	0.1	0.8	0.2	0	0	3.4
	9	Expo.	17.8	0.4	0	0.3	2.3	0.6	0	0	3.6
	16	Stat.	27.6	0	0	0	0	0	0	0	5.0
KL1C4	2	Lag	4.9	0	0	0.1	0.7	0	0	0	3.7
	9	Expo.	25.3	0.2	0	0.3	1.9	0.3	0	0	6.9
	16	Stat.	7.4	0	0	0	0.6	0.1	0	0	4.1
MAR1D2	2	Lag	3.3	0	0	0	0	0	0	0	nd
	9	Expo.	12.6	0	0	0	1.9	0	0	0	3.6
	16	Stat.	5.9	0	0	0	1.3	0	0	0	3.7

Supplementary Table S4. Weight-average molecular weight (Mw) and recovery yield of polysaccharides and proteins in the culture supernatants obtained for three *L. chlorophorum* strains (stationary phase): RCC1489, KL1C4 and MAR1D2 under non-axenic (NA) and pseudo-axenic (PA) conditions.

**Strain** Stationary phase	**Mw Polysaccharide (g/mol) (Recovery (%))**			**Mw Protein (g/mol) (Recovery (%))**		
	**Pic 1** 6.4-8.4 (min)	**Pic 2** 8.4-10.2 (min)	**Pic 3** 10.2-10.6 (min)	**Pic 1** 6.4-8.4 (min)	**Pic 2** 8.4-10.2 (min)	**Pic 3** 10.2-10.6 (min)
**RCC1489** (NA)	$9.953\ 10^{6}$ (12.5)	$1.252\ 10^{6}$ (6.9)	$3.320\ 10^{5}$ (3.3)	$1.110\ 10^{6}$ (1.5)	$1.757\ 10^{5}$ (1.1)	$5.979\ 10^{4}$ (0.08)
**RCC1489** (PA)	$6.271\ 10^{6}$ (10.0)	$4.746\ 10^{5}$ (7.1)	$4.239\ 10^{4}$ (13.0)	$6.211\ 10^{5}$ (1.2)	$1.399\ 10^{5}$ (5.0)	$3.612\ 10^{3}$ (0.7)
**KL1C4** (NA)	$1.076\ 10^{7}$ (9.4)	$2.290\ 10^{6}$ (7.0)	$5.119\ 10^{5}$ (6.9)	$1.549\ 10^{6}$ (1.5)	$3.885\ 10^{5}$ (1.4)	$1.239\ 10^{4}$ (0.2)
**KL1C4** (PA)	$2.535\ 10^{6}$ (1.3)	3.478×10^{5} (0.9)	$6.296\ 10^{3}$ (4.9)	$2.859\ 10^{5}$ (0.1)	$5.787\ 10^{5}$ (0.3)	$2.906\ 10^{3}$ (0.1)
**MAR1D2** (NA)	$7.169\ 10^{6}$ (9.3)	$2.954\ 10^{5}$ (21.4)	$7.246\ 10^{4}$ (9.2)	$9.778\ 10^{5}$ (1.5)	$3.943\ 10^{4}$ (2.7)	$4.824\ 10^{3}$ (0.3)
**MAR1D2** (PA)	$4.787\ 10^{6}$ (4.7)	$1.252\ 10^{5}$ (17.2)	$3.440\ 10^{4}$ (6.7)	$5.516\ 10^{5}$ (0.7)	$2.367\ 10^{4}$ (5.1)	$4.229\ 10^{3}$ (0.8)

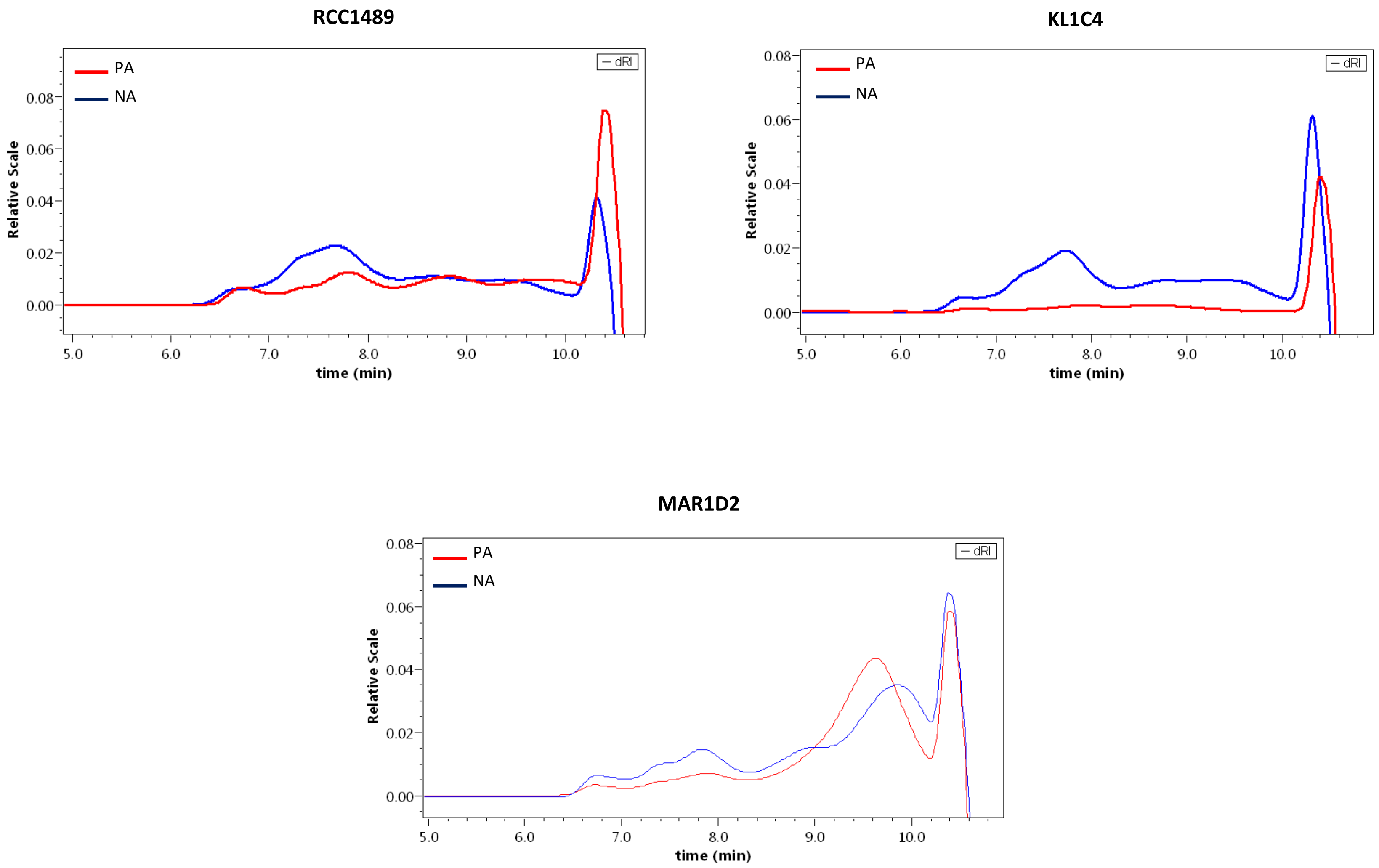

Supplementary Figure S1. HPSEC profiles (RI detectors) of culture supernatants obtained for three *L. chlorophorum* strains at stationary phase: RCC1489, KL1C4, MAR1D2 under non-axenic (NA) and pseudo-axenic (PA) conditions.

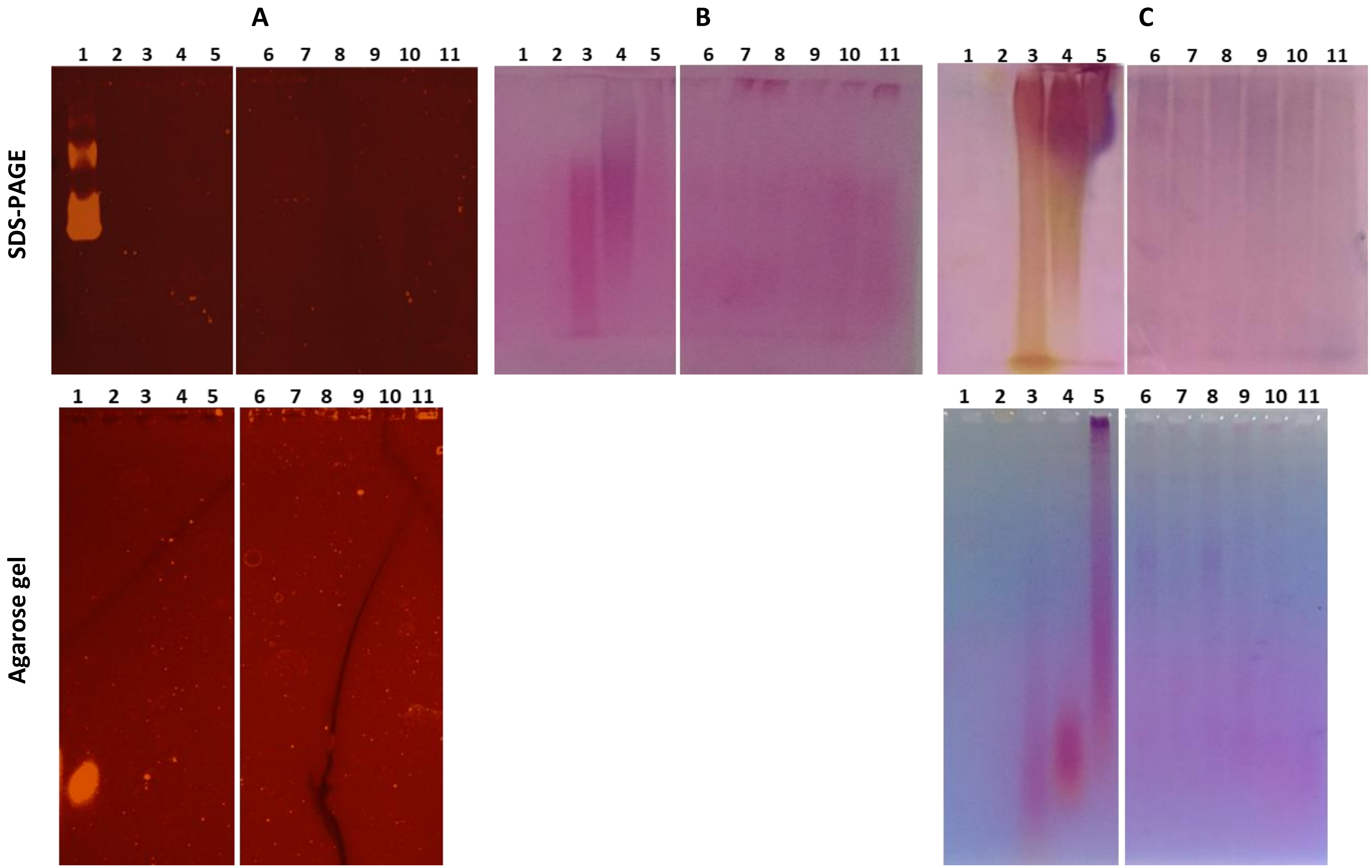

Supplementary Figure S2. Electrophoretic analysis of *L. chlorophorum* (RCC1489, KL1C4 and MAR1D2 strains) culture supernatants at the stationary phase under NA and PA conditions after SYPRO^{TM} Ruby (**A**), Schiff (**B**) and Stains All (**C**) staining. Lanes 1 to 5: references as follows, lane 1: Bovine Serum Albumin, lane 2: *E. coli* O111:B4 LPS, lane 3: Galactan sulphate 7.7% S, lane 4: Dextran sulphate sodium salt (MW 50 000, 16.0-19.0% S), lane 5: Dextran sulphate sodium salt (MW 500 000, 16.0-19.0% S). Lanes 6 and 7: RCC1489 under NA and PA conditions, lanes 8 and 9: KL1C4 under NA and PA conditions and lanes 10 and 11: MAR1D2 under NA and PA conditions.
